# Kidney age - chronological age difference (KCD) score provides an age-adapted measure of kidney function

**DOI:** 10.1186/s12882-021-02324-y

**Published:** 2021-04-26

**Authors:** Duncan J. Campbell, Jennifer M. Coller, Fei Fei Gong, Michele McGrady, Umberto Boffa, Louise Shiel, Danny Liew, Simon Stewart, Alice J. Owen, Henry Krum, Christopher M. Reid, David L. Prior

**Affiliations:** 1 St. Vincent’s Institute of Medical Research, 41 Victoria Parade, Fitzroy, Victoria 3065 Australia; 2 University of Melbourne, Parkville, Victoria Australia; 3 St. Vincent’s Hospital, Melbourne, Victoria Australia; 4 Royal Prince Alfred Hospital, Sydney, New South Wales Australia; 5 School of Medicine, University of Adelaide, Adelaide, South Australia Australia; 6 Public Health and Preventive Medicine, Monash University, Prahran, Victoria Australia; 7 Torrens University Australia, Adelaide, South Australia Australia; 8 School of Public Health, Curtin University, Bentley, Western Australia Australia

**Keywords:** Chronic kidney disease, eGFR, All-cause mortality, Cardiovascular disease

## Abstract

**Background:**

Given the age-related decline in glomerular filtration rate (GFR) in healthy individuals, we examined the association of all-cause death or cardiovascular event with the Kidney age - Chronological age Difference (KCD) score, whereby an individual’s kidney age is estimated from their estimated GFR (eGFR) and the age-dependent eGFR decline reported for healthy living potential kidney donors.

**Methods:**

We examined the association between death or cardiovascular event and KCD score, age-dependent stepped eGFR criteria (eGFRstep), and eGFR < 60 ml/min/1.73 m^2^ (eGFR60) in a community-based high cardiovascular risk cohort of 3837 individuals aged ≥60 (median 70, interquartile range 65, 75) years, followed for a median of 5.6 years.

**Results:**

In proportional hazards analysis, KCD score ≥ 20 years (KCD20) was associated with increased risk of death or cardiovascular event in unadjusted analysis and after adjustment for age, sex and cardiovascular risk factors. Addition of KCD20, eGFRstep or eGFR60 to a cardiovascular risk factor model did not improve area under the curve for identification of individuals who experienced death or cardiovascular event in receiver operating characteristic curve analysis. However, addition of KCD20 or eGFR60, but not eGFRstep, to a cardiovascular risk factor model improved net reclassification and integrated discrimination. KCD20 identified individuals who experienced death or cardiovascular event with greater sensitivity than eGFRstep for all participants, and with greater sensitivity than eGFR60 for participants aged 60–69 years, with similar sensitivities for men and women.

**Conclusions:**

In this high cardiovascular risk cohort aged ≥60 years, the KCD score provided an age-adapted measure of kidney function that may assist patient education, and KCD20 provided an age-adapted criterion of eGFR-related increased risk of death or cardiovascular event. Further studies that include the full age spectrum are required to examine the optimal KCD score cut point that identifies increased risk of death or cardiovascular event, and kidney events, associated with impaired kidney function, and whether the optimal KCD score cut point is similar for men and women.

**Trial registration:**

ClinicalTrials.gov NCT00400257, NCT00604006, and NCT01581827.

**Supplementary Information:**

The online version contains supplementary material available at 10.1186/s12882-021-02324-y.

## Background

Chronic kidney disease (CKD) “is defined as abnormalities of kidney structure or function, present for >3 months, with implications for health” [1], which include end-stage kidney disease, but predominantly premature mortality and cardiovascular (CV) events. Criteria for the definition of CKD in adults are: (1) signs of kidney damage, most often determined by an elevated urine albumin (or protein)- to-creatinine ratio; or (2) reduced kidney function, indicated by glomerular filtration rate (GFR) < 60 ml/min/1.73 m^2^ [1]. However, the GFR cut point of 60 ml/min/1.73 m^2^, based on a meta-analysis of the relationship between estimated GFR (eGFR) and mortality and morbidity in approximately 1.5 million participants from the general population [2], does not take account of the normal age-related decline in eGFR [3–9], and is subject to ongoing debate [1–3, 10–18]. Two potential limitations of the eGFR cut point of 60 ml/min/1.73 m^2^ are (i) its failure to identify individuals with eGFR-related increased risk of death or CV event who have eGFR above this cut point, and (ii) its overdiagnosis of CKD among individuals with eGFR below the cut point because of normal age-related decline in eGFR. The first limitation is illustrated by the association of eGFR 60–74 ml/min/1.73 m^2^ with increased risk of death and CV event in individuals aged < 65 years [19, 20].

**Fig. 1:**
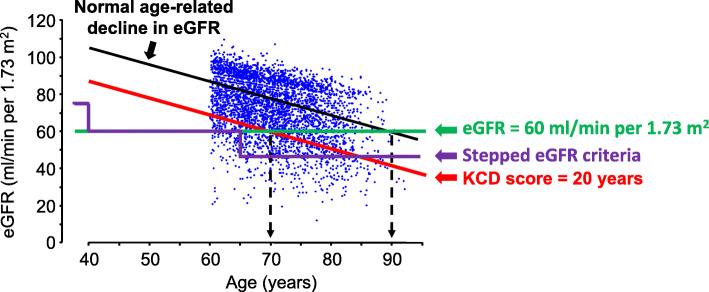
Scattergram plot of eGFR in relation to age for 3837 SCREEN-HF participants. The black line represents the age-related decline in eGFR from 105 ml/min/1.73 m^2^ at age 40 years to 60 ml/min/1.73 m^2^ at age 90 years in healthy living potential kidney donors [3]. eGFR values below the black line represent eGFR values below that of a healthy living potential kidney donor of the same age, and correspond to a kidney age that exceeds the chronological age. A 70-year old individual with an eGFR of 60 ml/min/1.73 m^2^ has an eGFR of a healthy 90-year-old; thus, the kidney age is 20 years older than the chronological age, and the Kidney age - Chronological age Difference (KCD) score is 90–70 = 20 years. Whereas CKD may be defined by eGFR < 60 ml/min/1.73 m^2^ (green line), age-dependent stepped eGFR criteria of Delanaye et al. [10] define CKD as eGFR < 75 ml/min/1.73 m^2^ for age < 40 years, < 60 ml/min/1.73 m^2^ for individuals between 40 and 65 years, and < 45 ml/min/1.73 m^2^ for age > 65 years (purple line) The red line corresponds to a KCD score of 20 years

An age-adapted definition of CKD has been proposed by several authors [7, 10, 21, 22] and age-dependent reference intervals for eGFR reported [7–9], but the relationship between these reference intervals and health outcomes has not been examined. Based on an analysis of all-cause mortality according to categories of eGFR and age in patients with little or no albuminuria [4], Delanaye et al. recently proposed age-dependent stepped eGFR criteria for CKD diagnosis, whereby CKD was defined by eGFR < 75 ml/min/1.73 m^2^ for individuals aged < 40 years, < 60 ml/min/1.73 m^2^ for individuals aged 40–65 years, and < 45 ml/min/1.73 m^2^ for individuals aged > 65 years (Fig. 1) [10]. However, these stepped eGFR criteria may fail to identify individuals with eGFR-related increased risk of death aged 40–64 years with eGFR ≥60 ml/min/1.73 m^2^, or aged 65–74 years with eGFR ≥45 ml/min/1.73 m^2^, described by Hallan et al. [20].

We propose an alternative age-adapted measure of kidney function, the Kidney age - Chronological age Difference (KCD) score, whereby an individual’s kidney age is estimated from their eGFR and the age-dependent eGFR decline based on the metanalysis of 5482 healthy living potential kidney donors [3]. In this metanalysis, mean GFR of healthy living potential kidney donors was 104.9 ml/min/1.73 m^2^ at age 35 years, and declined at 0.89 ml/min/1.73 m^2^ per year after age 40 years [3]. To assist description of the KCD score and its calculation, we assumed a mean eGFR of 105 ml/min/1.73 m^2^ at age 40 years, and decline of 0.9 ml/min/1.73 m^2^ per year after age 40 years; from this relationship, expected eGFR for a healthy individual was calculated for their known chronological age:

1 $$ \mathrm{eGFR}=105-\left[0.9\ast \left(\mathrm{age}\ \mathrm{in}\ \mathrm{years}-40\right)\right] $$

This equation calculates an eGFR for a healthy 90-year old individual to be 60 ml/min/1.73 m^2^. Equation (1) can be transposed to give the kidney age corresponding to a measured eGFR, assuming an eGFR of 105 ml/min/1.73 m^2^ at age 40 years and decline in eGFR of 0.9 ml/min/1.73 m^2^ per year after age 40 years for a healthy individual:

2 $$ \mathrm{Kidney}\ \mathrm{age}\ \mathrm{in}\ \mathrm{years}=\left[\left(1/0.9\right)\ast \left(105-\mathrm{eGFR}\right)\right]+40 $$

An eGFR that falls below the regression line for the age-related decline in eGFR of a healthy individual (Fig. 1) indicates a kidney age that exceeds its chronological age. Thus, if a 70-year old individual has an eGFR of 60 ml/min/1.73 m^2^, their eGFR corresponds to a kidney age of 90 years for a healthy individual, and their KCD score is 90–70 = 20 years. This concept of kidney age is analogous to the concept of heart age, described by D’Agostino et al. [23].

We compared the associations of all-cause death or CV event with the KCD score, the age-dependent stepped eGFR criteria of Delanaye et al. [10] (eGFRstep), and the eGFR cut point of 60 ml/min/1.73 m^2^ (eGFR60) in a prospective cohort study of 3837 community-based individuals at increased CV event risk who had eGFR measured at baseline. Our hypothesis was that the KCD score would be superior to eGFRstep in identifying individuals at increased risk of death or CV event.

## Methods

### Study population

The SCReening Evaluation of the Evolution of New Heart Failure (SCREEN-HF) study, a prospective cohort study of men and women recruited from the community, has been described elsewhere [24–27]. A CONSORT flow diagram describing participant recruitment and follow-up is shown in Supplementary Fig. 1. In summary, 44,000 members of private health fund Bupa, resident in Melbourne or Shepparton, Victoria, Australia, were invited to participate. Inclusion criteria were age ≥ 60 years with one or more of self-reported treatment for hypertension or diabetes for ≥2 years, myocardial infarction or other ischaemic heart disease, valvular heart disease, irregular or rapid heart rhythm, cerebrovascular disease, or kidney impairment. We excluded individuals with previously diagnosed heart failure and those with well-recognised risk for heart failure, such as previous valve surgery or documented valve abnormality graded >mild, left ventricular ejection fraction < 50% or other known cardiac abnormality on previous echocardiography or other cardiac imaging. Recruitment commenced in May 2007 and was completed in January 2010. Of the 4054 individuals enrolled at the baseline visit (Visit 1), 3847 met the inclusion and exclusion criteria, of whom 3837 had eGFR measurement and complete baseline CV risk factor data. The SCREEN-HF study was registered at ClinicalTrials.gov NCT00400257, NCT00604006, and NCT01581827.

Details of the collection of baseline data are described elsewhere [24–27]. Serum creatinine was measured using the Siemens CRE_2 Jaffe kinetic method on the Siemens Advia 2400, a method that is IDMS traceable. eGFR was calculated using the Chronic Kidney Disease Epidemiology Collaboration (CKD-EPI) equation [28].

### Outcome assessment

Follow-up was by participant visits and phone follow-up (Supplementary Fig. 1). All participant files were reviewed by a CV physician and documentation of all deaths and CV events was requested from hospitals, and the participant’s primary care provider, physician and cardiologist. Adjudicated heart failure diagnosis according to European Society of Cardiology criteria of 2012 has been described previously [25, 29]. Diagnoses of myocardial infarction, stroke and transient ischaemic attack were based on published criteria [30, 31].

### Statistical analysis

Continuous variables were summarised as medians (interquartile range, IQR) and categorical variables summarised as numbers (percentages). Study outcome was death or CV event (incident myocardial infarction, heart failure, stroke/transient ischaemic attack, and coronary revascularisation). Data were censored at the date of last contact. Sensitivities and specificities for identification of individuals who experienced a study outcome were compared using McNemar’s test with Yates correction. Hazard ratios (HRs) and Bier scores, with 95% confidence interval (CI), for death or CV event were calculated from unadjusted proportional hazards models and also after adjustment for age, sex and CV risk factors (previous myocardial infarction, coronary revascularisation, stroke, transient ischaemic attack, peripheral vascular disease, diabetes, atrial fibrillation (AF), log_2_(body mass index, BMI), systolic blood pressure, antihypertensive medication, and smoking status on enrolment). Serum lipids, measured on blood collected at Visit 2 (Supplementary Fig. 1), were available for 3068 participants. However, neither total cholesterol nor high density lipoprotein cholesterol was statistically significantly associated with death or CV event in multivariable proportional hazards analysis; therefore, total and high density lipoprotein cholesterol were not included in the CV risk factor model, and CV risk factor data were available for 3837 participants. Inspection of Schoenfeld residuals confirmed that proportional hazards assumptions were satisfied. Area under the curve (AUC) statistics were estimated from time-dependent receiver operating characteristic (ROC) curves for 5-year follow-up with asymptotic CIs as described by Blanche et al. [32]. Continuous net reclassification improvement (NRI) for time-to-event data with inverse probability weighting was calculated as described by Pencina et al. [33–35] and CIs estimated with bootstrap resampling (*n* = 1000). Integrated discrimination improvement (IDI) and calibration plots were calculated using sex-specific 5-year absolute risk derived from models based on CV risk factors alone, and from CV risk factor models to which KCD score ≥ 20 years (KCD20), eGFRstep or eGFR60 were added, using coefficients from multivariable proportional hazards models and Kaplan-Meier estimates of baseline event rate, as described by Goff et al. [36]. IDI was calculated as described by Pencina et al. [33]. Given that absolute risk models were based on Kaplan-Meier estimates of baseline event rate, calibration plots were constructed comparing mean sex-specific 5-year absolute risk of death or CV event with mean Kaplan-Meier estimates of observed 5-year risk for deciles of absolute risk. A two-sided *P* value < 0.05 was considered to indicate statistical significance. Analyses were conducted using Statview 5.0.1 (SAS Institute, Cary, NC, USA), and R version 4.0.2.

## Results

### Characteristics of study population

Baseline participant characteristics are shown in Table 1. Median age was 70 (IQR: 65, 75) years on enrolment, 55% were male, 86% had hypertension, 18% had diabetes, 32% were obese (BMI ≥30 kg/m^2^), 45% were overweight (25 < BMI < 30 kg/m^2^), 22% had a history of ischaemic heart disease, 10% had a previous myocardial infarction, and 10% had AF.

**Table 1:** Characteristics of 3837 SCREEN-HF participants who had eGFR measurement and complete data for cardiovascular risk factors on enrolment

Characteristic	Men	Women
	*n* = 2096	*n* = 1741
Age (years)	70 (65, 75)	70 (65, 75)
Bupa member	1921 (92%)	1598 (92%)
Systolic blood pressure (mmHg)	141 (131, 153)	137 (127, 151)
Diastolic blood pressure (mmHg)	81 (75, 88)	80 (73, 87)
Pulse pressure (mmHg)	60 (52, 70)	57 (49, 68)
Heart rate (bpm)	69 (61, 77)	72 (65, 80)
Body mass index (kg/m^2^)	28 (25, 31)	28 (25, 32)
Waist circumference (cm)	103 (96, 110)	94 (86, 103)
Cardiovascular risk factors		
Hypertension	1716 (82%)	1571 (90%)
Diabetes	429 (20%)	272 (16%)
Obesity (BMI ≥30 kg/m^2^)	622 (30%)	615 (35%)
Overweight (25 > BMI < 30 kg/m^2^)	1049 (50%)	682 (39%)
eGFR < 60 ml/min/1.73 m^2^	411 (20%)	373 (21%)
Previous myocardial infarction	302 (14.4%)	87 (5.0%)
Coronary revascularisation	460 (21.9%)	115 (6.6%)
Total ischaemic heart disease	607 (29%)	243 (14%)
Previous stroke or transient ischaemic attack	250 (11.9%)	168 (9.6%)
Peripheral vascular disease	93 (4.4%)	32 (1.8%)
Cardiovascular disease	810 (39%)	394 (23%)
Atrial fibrillation	240 (11.5%)	151 (8.7%)
Pacemaker	47 (2.2%)	18 (1.0%)
Obstructive sleep apnoea	215 (10.3%)	63 (3.6%)
Physical inactivity	1115 (63%)	1013 (71%)
Tobacco use		
Current smoker	79 (3.8%)	59 (3.4%)
Former smoker	1161 (55%)	587 (34%)
Non-smoker	856 (41%)	1094 (63%)
Alcohol > 2 drinks/day	616 (29.4%)	144 (8.3%)
Medication use		
Antihypertensive therapy	1873 (89%)	1613 (93%)
ß-blocker	477 (23%)	411 (24%)
ACE inhibitor	715 (34%)	471 (27%)
ARB	912 (44%)	888 (51%)
ACE inhibitor and/or ARB	1549 (74%)	1295 (74%)
Calcium channel blocker	570 (27%)	477 (27%)
Statin therapy	1159 (55%)	830 (48%)
Thiazide diuretic	616 (29%)	563 (32%)
Loop diuretic	47 (2.2%)	62 (3.6%)
Mineralocorticoid antagonist	9 (0.4%)	14 (0.8%)
Digoxin therapy	65 (3.1%)	32 (1.8%)
Aspirin therapy	976 (47%)	624 (36%)
Clopidogrel therapy	145 (6.9%)	94 (5.4%)
Warfarin therapy	120 (5.7%)	57 (3.3%)
NSAID therapy	170 (8.1%)	152 (8.7%)
Insulin therapy	67 (3.2%)	41 (2.4%)
Oral anti-diabetic medication	298 (14%)	178 (10%)
Nitrate therapy	109 (5.2%)	108 (6.2%)
Biochemistry and haematology		
eGFR (ml/min/1.73 m^2^)	76 (63, 86)	74 (62, 86)
Haemoglobin (g/dL)	14.6 (13.8, 15.3)	13.3 (12.7, 14.0)
White cell count (×10^9^/L)	7.1 (6.1, 8.2)	7.1 (6.1, 8.3)
Platelets (×10^9^/L)	212 (183, 246)	250 (216, 288)
Total cholesterol (mmol/L)	4.5 (3.8, 5.2)	5.2 (4.5, 5.9)
High density lipoprotein cholesterol (mmol/L)	1.1 (0.9, 1.4)	1.4 (1.2, 1.7)

Data shown as median (interquartile range) or n (%). *ACE* angiotensin converting enzyme, *ARB* angiotensin II type 1 receptor blocker, *bpm* beats per minute, *eGFR* estimated glomerular filtration rate calculated using the Chronic Kidney Disease Epidemiology Collaboration (CKD-EPI) equation [28], *NSAID* non-steroidal anti-inflammatory drug. Total ischaemic heart disease refers to myocardial infarction, coronary revascularisation, coronary artery disease detected on coronary angiography, and angina. Cardiovascular disease refers to total ischaemic heart, cerebrovascular and peripheral vascular disease. Physical activity was assessed using the New York Heart Association questionnaire [37], and physical inactivity refers to participants who did not walk for, on average, ≥30 min per day and/or participate in, on average, ≥10 min per day of more vigorous exercise, including housework, for the 1757 men and 1419 women who completed the questionnaire. Alcohol > 2 drinks/day refers to consumption of more than 2 standard drinks on any day [38]. The numbers receiving antihypertensive therapy exceeded the numbers with hypertension because participants without hypertension received antihypertensive therapy. Total cholesterol and high-density lipoprotein cholesterol were measured in 1712 men and 1356 women. Data for cardiovascular disease, diabetes, obstructive sleep apnoea, smoking, alcohol intake and drug therapy were from self-report

Median follow-up was 5.6 (IQR: 4.6, 6.3) years. Among participants who experienced death or CV event, median time to death or CV event was 3.4 (IQR: 2.0, 5.0) years; 782 participants experienced death or CV event during follow-up, and 585 experienced death or CV event during 5 years of follow-up (Supplementary Table 1).

### Relationship of eGFR to age

The relationship between participant age and baseline eGFR is shown in Fig. 1; the regression line for age-related decline in eGFR according to the meta-analysis for healthy living potential kidney donors reported by Pottel et al. [3], and the corresponding regression line for a KCD score of 20 years, are shown. Also shown are the stepped eGFR criteria [10], and the eGFR cut point of 60 ml/min/1.73 m^2^ for CKD definition [1].

### Distribution of eGFR and KCD scores

**Fig. 2:**
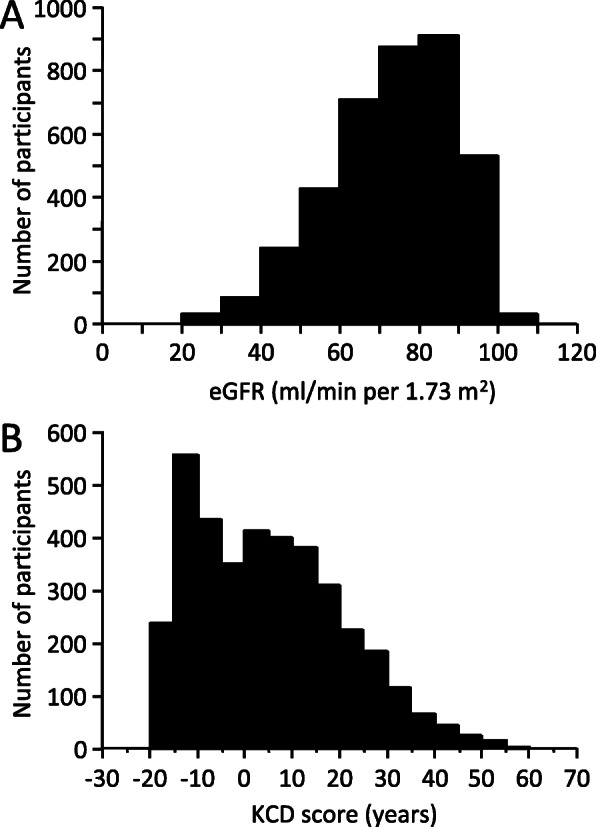
Histograms of the numbers of participants according to eGFR (**a**) and KCD score (**b**)

eGFR had a skewed distribution and 784 participants (20%) had an eGFR < 60 ml/min/1.73 m^2^ (Fig. 2a). KCD scores also had a skewed distribution (Fig. 2b). A negative KCD score represented participants with eGFR above the regression line for healthy living potential kidney donors, and only 68 (1.8%) participants had a KCD score < − 20 years. By contrast, 694 (18.1%) participants had a KCD score ≥ 20 years (KCD20) and 468 (12.2%) had a KCD score ≥ 25 years.

### Relationship of KCD score to BMI

BMI was similar for KCD score categories ranging from <− 20 to ≥30 years (Supplementary Fig. 2), with no evidence that extremes of KCD score were associated with extreme deviation of body composition that may have impacted on serum creatinine, eGFR or KCD score.

### Associations with death or CV event

**Fig. 3:**
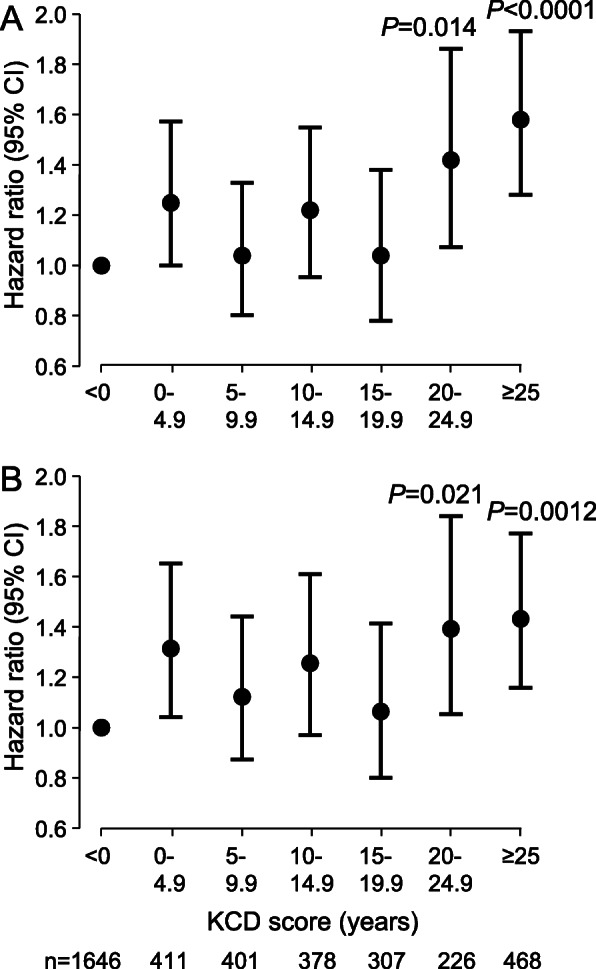
Hazard ratios for death or CV event according to KCD score. Hazard ratios (95% confidence interval, CI) for KCD score alone (**a**) or adjusted (**b**) for age, sex, and CV risk factors (previous myocardial infarction, coronary revascularisation, stroke or transient ischaemic attack, peripheral vascular disease, diabetes, atrial fibrillation, log_2_(BMI), systolic blood pressure, antihypertensive medication, and smoking status on enrolment). Hazard ratios from proportional hazards analysis of all events during follow-up (*n* = 782)

In proportional hazards analysis, with participants with KCD scores < 0 years as the reference group, KCD scores 20 - < 25 years and ≥ 25 years were associated with increased risk of death or CV event in unadjusted analysis (Fig. 3a), and after adjustment for age, sex and CV risk factors (Fig. 3b). KCD score ≥ 20 years (KCD20) alone, eGFRstep alone, and eGFR60 alone were each associated with risk of death or CV event in unadjusted (univariate) proportional hazards analysis (Tables 2, 3). Moreover, KCD20 and eGFR60, but not eGFRstep, were each associated with risk of death or CV event in adjusted (multivariable) analyses that included CV risk factors (Tables 2, 3).

**Table 2:** Proportional hazards analysis of 5-year association of death or cardiovascular event with cardiovascular risk factors alone, with KCD20 alone, and with KCD20 together with cardiovascular risk factors

Variable	Cardiovascular risk factors alone		KCD20 alone or KCD20 with cardiovascular risk factors	
	Hazard ratio (95% CI)	*P*	Hazard ratio (95% CI)	*P*
Univariate analysis				
KCD20			1.44 (1.19, 1.74)	0.0004
Multivariable analysis				
Age (years)	1.07 (1.05, 1.08)	< 0.0001	1.07 (1.05, 1.08)	< 0.0001
Male sex	1.63 (1.35, 1.97)	< 0.0001	1.63 (1.35, 1.97)	< 0.0001
Systolic blood pressure (per 10 mmHg)	1.05 (1.00, 1.11)	0.037	1.06 (1.01, 1.11)	0.028
Antihypertensive medication	0.93 (0.70, 1.22)	0.58	0.89 (0.68, 1.18)	0.43
Diabetes	1.56 (1.29, 1.90)	< 0.0001	1.54 (1.27, 1.87)	< 0.0001
Log_2_(body mass index, kg/m^2^)	1.80 (1.23, 2.65)	0.0026	1.74 (1.18, 2.56)	0.0050
Myocardial infarction	0.96 (0.74, 1.24)	0.74	0.96 (0.74, 1.24)	0.74
Coronary revascularisation	1.75 (1.40, 2.19)	< 0.0001	1.75 (1.40, 2.19)	< 0.0001
Previous stroke or transient ischaemic attack	1.29 (1.01, 1.65)	0.041	1.27 (1.00, 1.63)	0.053
Peripheral vascular disease	1.51 (1.06, 2.16)	0.022	1.50 (1.05, 2.13)	0.025
Atrial fibrillation/flutter	1.62 (1.29, 2.03)	< 0.0001	1.64 (1.31, 2.06)	< 0.0001
Current or former smoker	1.21 (1.02, 1.44)	0.029	1.22 (1.03, 1.45)	0.023
KCD20			1.31 (1.08, 1.60)	0.0076

*CI* confidence interval, *KCD20* Kidney age-Chronological age Difference score ≥ 20 years

**Table 3:** Proportional hazards analysis of 5-year association of death or cardiovascular event with eGFRstep or eGFR60 alone, and with eGFRstep or eGFR60 together with cardiovascular risk factors

Variable	eGFRstep alone or eGFRstep with cardiovascular risk factors		eGFR60 alone or eGFR60 with cardiovascular risk factors	
	Hazard ratio (95% CI)	*P*	Hazard ratio (95% CI)	*P*
Univariate analysis				
eGFRstep	1.78 (1.37, 2.31)	< 0.0001		
eGFR60			1.84 (1.54, 2.19)	< 0.0001
Multivariable analysis				
Age (years)	1.07 (1.05, 1.08)	< 0.0001	1.06 (1.05, 1.08)	< 0.0001
Male sex	1.63 (1.35, 1.97)	< 0.0001	1.65 (1.36, 1.99)	< 0.0001
Systolic blood pressure (per 10 mmHg)	1.05 (1.00, 1.11)	0.034	1.06 (1.01, 1.11)	0.022
Antihypertensive medication	0.91 (0.69, 1.21)	0.52	0.89 (0.67, 1.17)	0.39
Diabetes	1.54 (1.26, 1.87)	< 0.0001	1.54 (1.27, 1.87)	< 0.0001
Log_2_(body mass index, kg/m^2^)	1.77 (1.21, 2.61)	0.0036	1.71 (1.16, 2.52)	0.0066
Myocardial infarction	0.96 (0.74, 1.24)	0.74	0.96 (0.74, 1.24)	0.76
Coronary revascularisation	1.75 (1.40, 2.19)	< 0.0001	1.74 (1.39, 2.17)	< 0.0001
Previous stroke or transient ischaemic attack	1.27 (1.00, 1.63)	0.053	1.27 (0.99, 1.62)	0.057
Peripheral vascular disease	1.51 (1.06, 2.15)	0.022	1.49 (1.05, 2.12)	0.026
Atrial fibrillation/flutter	1.63 (1.30, 2.05)	< 0.0001	1.65 (1.31, 2.06)	< 0.0001
Current or former smoker	1.22 (1.02, 1.44)	0.027	1.22 (1.03, 1.45)	0.022
eGFRstep	1.29 (0.98, 1.70)	0.067		
eGFR60			1.37 (1.13, 1.65)	0.0015

*CI* confidence interval, *eGFR60* eGFR < 60 ml/min/1.73 m^2^, *eGFRstep* age-dependent stepped eGFR criteria of Delanaye et al. [10]

**Fig. 4:**
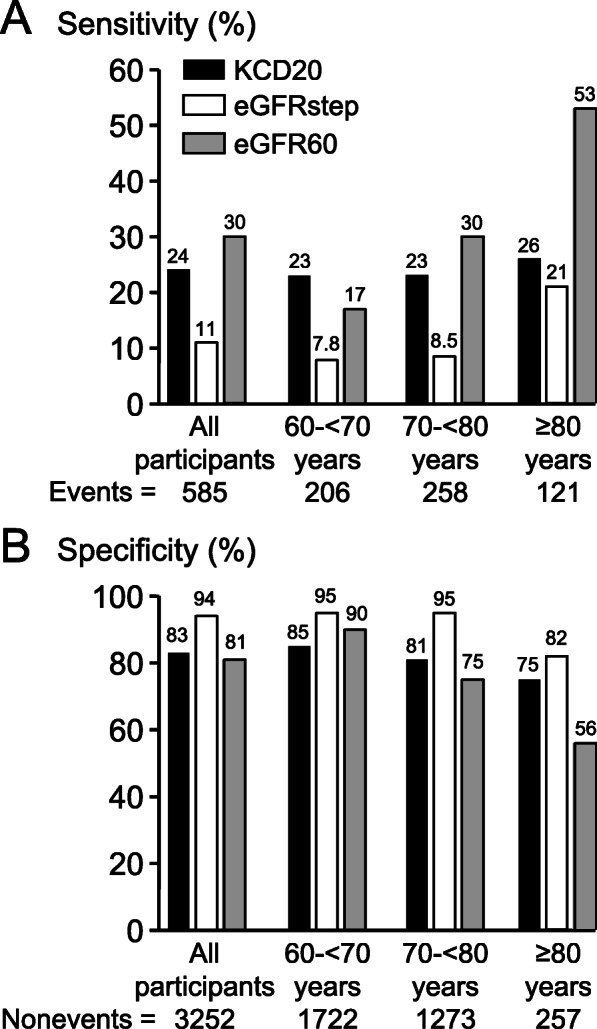
Sensitivity (**a**) and specificity (**b**) for the identification of individuals who experienced death or CV event during 5-year follow-up. Sensitivity and specificity for either Kidney age - Chronological age Difference score ≥ 20 years (KCD20), age-dependent stepped eGFR criteria of Delanaye et al. [10] (eGFRstep), or eGFR < 60 ml/min/1.73 m^2^ (eGFR60), for all participants, and for participants aged 60- < 70 years, 70- < 80 years, and ≥ 80 years. Among all participants, and each age category, sensitivities and specificities, compared using McNemar’s test with Yates correction, were statistically significantly different between KCD20, eGFRstep and eGFR60 (*P* < 0.05)

KCD20 identified individuals who experienced death or CV event with greater sensitivity than eGFRstep for all participants (*P* < 0.0001), and in separate analysis of participants aged 60–69 years (*P* < 0.0001), 70–79 years (*P* < 0.0001), and ≥ 80 years (*P* < 0.046) (Fig. 4a). KCD20 also identified individuals who experienced death or CV event with greater sensitivity than eGFR60 for participants aged 60–69 years (*P* < 0.0001) (Fig. 4a). The higher sensitivity for identification of individuals who experienced death or CV event by eGFR60 for participants ≥70 years of age was associated with progressive decrease in specificity (Fig. 4b). By contrast, the lower sensitivity for identification of individuals who experienced death or CV event by eGFRstep was associated with higher specificity (Fig. 4b).

Sensitivities and specificities for the identification of individuals who experienced death or CV event by KCD20, eGFRstep and eGFR60 were similar for men and women (Supplementary Table 2).

### Discrimination and classification

In ROC curve analysis, AUC for eGFR60 alone was higher than for either KCD20 alone (*P* < 0.0001) or eGFRstep alone (*P* < 0.0001), with no statistically significant difference between the AUC for KCD20 alone and eGFRstep alone (Table 4). The multivariable proportional hazards model based on CV risk factors alone identified individuals who experienced death or CV event with AUC of 0.717 (95% CI: 0.694, 0.740). No improvement in AUC was obtained when KCD20, eGFRstep, or eGFR60 was added to the CV risk factor model (Table 4). Addition of KCD20 or eGFR60 to the CV risk factor model decreased NRI for events, but increased NRI for non-events, such that the NRI for combined events and non-events was increased when KCD20 or eGFR60 was added to the CV risk factor model, whereas addition of eGFRstep to the CV risk factor model did not change NRI (Table 5). Moreover, addition of KCD20 or eGFR60, but not eGFRstep, to the CV risk factor model improved IDI (Table 5).

**Table 4:** Time-dependent receiver operating characteristic curve analysis of identification of individuals who experienced death or cardiovascular event by cardiovascular risk factors, by KCD20, eGFRstep or eGFR60 alone, and by the combination of cardiovascular risk factors and KCD20, eGFRstep or eGFR60

Model	AUC (95% CI)
Cardiovascular risk factors alone	0.717 (0.694, 0.740)^a^
KCD20 alone	0.531 (0.512, 0.550)^b^
eGFRstep alone	0.523 (0.510, 0.537)^b^
eGFR60 alone	0.561 (0.541, 0.581)^b^
Cardiovascular risk factors and KCD20	0.719 (0.696, 0.742)^a^
Cardiovascular risk factors and eGFRstep	0.718 (0.695, 0.741)^a^
Cardiovascular risk factors and eGFR60	0.718 (0.695, 0.741)^a^

*AUC* area under the curve calculated from time-dependent receiver operating characteristic curve analysis for 5-year follow-up [32], *CI* confidence interval, *eGFR60* eGFR < 60 ml/min/1.73 m^2^, *eGFRstep* age-dependent stepped eGFR criteria of Delanaye et al. [10], *KCD20* Kidney age-Chronological age Difference score ≥ 20 years

The model with cardiovascular risk factors alone included age, sex, previous myocardial infarction, coronary revascularisation, stroke or transient ischaemic attack, peripheral vascular disease, diabetes, atrial fibrillation, log_2_(BMI), systolic blood pressure, antihypertensive medication, and smoking status on enrolment

^a^There were no statistically significant differences between AUC for the model with cardiovascular risk factors alone, and the models with cardiovascular risk factors and KCD20, eGFRstep, or eGFR60

^b^AUC for eGFR60 alone was higher than for KCD20 alone (*P* < 0.0001) and for eGFRstep alone (*P* = 0.0001), with no statistically significant difference between AUC for KCD20 alone and for eGFRstep alone (*P* = 0.31)

**Table 5:** Continuous net reclassification improvement (NRI) and integrated discrimination improvement (IDI) for the identification of individuals who experienced death or cardiovascular event during 5-year follow-up by cardiovascular risk factor models including either KCD20, eGFRstep, or eGFR60, in comparison with cardiovascular risk factor model without kidney function parameter

	Estimate (95% CI)	*P* value
Cardiovascular risk factor model including KCD20, in comparison with cardiovascular risk factor model without kidney function parameter.		
NRI (events & non-events)	0.126 (0.042, 0.201)	0.0020
NRI (events)	−0.504 (−0.575, − 0.424)	< 0.0001
NRI (non-events)	0.630 (0.580, 0.662)	< 0.0001
IDI	0.00359 (0.00098, 0.00620)	0.0069
Cardiovascular risk factor model including eGFRstep, in comparison with cardiovascular risk factor model without kidney function parameter.		
NRI (events & non-events)	−0.010 (− 0.163, 0.122)	NS
NRI (events)	−0.607 (− 0.705, 0.599)	NS
NRI (non-events)	0.597 (−0.485, 0.755)	NS
IDI	0.00174 (−0.00024, 0.00372)	NS
Cardiovascular risk factor model including eGFR60, in comparison with cardiovascular risk factor model without kidney function parameter.		
NRI (events & non-events)	0.206 (0.105, 0.295)	< 0.0001
NRI (events)	−0.329 (− 0.401, − 0.235)	< 0.0001
NRI (non-events)	0.534 (0.447, 0.576)	< 0.0001
IDI	0.00603 (0.00259, 0.00947)	0.0006

*CI* confidence interval, *eGFR60* eGFR < 60 ml/min/1.73 m^2^, *eGFRstep* age-dependent stepped eGFR criteria of Delanaye et al. [10], *KCD20* Kidney age-Chronological age Difference score ≥ 20 years

Continuous NRI for time-to-event data with inverse probability weighting calculated as described by Pencina et al. [33–35] and CI estimated with bootstrapping. IDI calculation based on sex-specific 5-year absolute risk, calculated as described by Pencina et al. [33]

Calibration plots showed reasonable agreement between model-based calculated 5-year absolute risk and observed 5-year risk for models based on CV risk factors alone, and models that included KCD20, eGFRstep or eGFR60 (Supplementary Fig. 3). Brier scores were not different between proportional hazards models with CV risk factors alone, or with addition of KCD20, eGFRstep or eGFR60 to the CV risk factor model (Supplementary Fig. 3).

## Discussion

The challenge for a CKD definition based on an eGFR threshold “with implications for health” [1] is to differentiate changes in kidney structure and function associated with premature morbidity and mortality from the anatomic and functional changes in the kidneys observed with healthy, normal aging [6, 16], a challenge that has stimulated investigation of age-adapted CKD criteria [7, 10, 21, 22]. The KCD score provided a continuous age-adapted measure of kidney function. Moreover, the association of KCD score ≥ 20 years (KCD20) with increased eGFR-related risk of death or CV event provided an age-adapted criterion for CKD that was able to diagnose CKD in individuals aged < 70 years with eGFR ≥60 ml/min/1.73 m^2^, and avoided the diagnosis of CKD in those with eGFR < 60 ml/min/1.73 m^2^ due to age. KCD20 had higher sensitivity than eGFRstep in identifying individuals aged ≥60 years with increased risk of death or CV event, and higher sensitivity than eGFR60 in identifying individuals aged 60–69 years with increased risk of death or CV event. Moreover, KCD20 was superior to eGFRstep with respect to improvement in NRI and IDI for identification of individuals with increased risk of death or CV event.

Our use of KCD20 as a criterion for diagnosis of CKD was based on proportional hazards analysis of the risk of death or CV event in SCREEN-HF participants, who were ≥ 60 years of age. Further studies that include the full age spectrum are required to examine the optimal KCD score cut point that identifies eGFR-related increased risk of death or CV event, and whether the optimal KCD score cut point is similar for men and women. Nevertheless, our use of KCD20 as a criterion for diagnosis of CKD was supported by the meta-analysis of ~ 2 million individuals by Hallan et al. who reported that individuals aged 18–54 and 55–64 years with eGFR 60–74 ml/min/1.73 m^2^ had increased risk for all-cause mortality in comparison with individuals with eGFR of 75–89 ml/min/1.73 m^2^, and also by the meta-analysis of 637,315 individuals by Matsushita et al. who reported that individuals with eGFR 60–74 ml/min/1.73 m^2^ had increased risk for CV mortality, coronary heart disease and heart failure, in comparison with individuals with eGFR of 95 ml/min/1.73 m^2^ [19, 20].

The meta-analyses of Hallan et al. and Matsushita et al. showed a J-shaped association of high eGFR with increased relative and absolute mortality risk in patients older than 55 years [20], and with increased cardiovascular mortality, coronary heart disease and stroke [19]. Hallan et al. [20] proposed this J-shaped association was caused by the influence of patients with reduced muscle mass due to malnutrition and other effects associated with cancer or other significant comorbidities. In our study of SCREEN-HF participants we found no evidence that low KCD score (<− 20 years) was associated with malnutrition. However, much larger studies of the full age spectrum in general populations are required to examine whether a J-shaped association exists for the KCD score and death and CV outcomes.

The higher sensitivity for identification of individuals with increased risk of death or CV event by KCD20, in comparison with eGFRstep and eGFR60, in SCREEN-HF participants aged 60–69 years was due to its identification of increased risk of death or CV event in individuals < 70 years of age with eGFR > 60 ml/min/1.73 m^2^, whereas neither eGFRstep nor eGFR60 was able to identify individuals aged > 40 years with eGFR > 60 ml/min/1.73 m^2^ and eGFR-related increased risk of death or CV event. The higher sensitivity for identification of individuals with increased risk of death or CV event by KCD20 in participants aged 60–69 years was in agreement with the meta-analyses of Hallan et al. [20] and Matsushita et al. [19], described above. The importance of identifying individuals < 70 years of age with eGFR > 60 ml/min/1.73 m^2^ with eGFR-related increased risk of death or CV event is their potential to benefit from early recognition of their increased risk, and from intervention to slow the rate of decline in their kidney function and decrease their risk.

eGFR60 identified individuals with increased risk of death or CV event with higher sensitivity than KCD20 in participants ≥70 years of age because eGFR60, in contrast to KCD20, was not adjusted for age, and the proportion of participants with eGFR < 60 ml/min/1.73 m^2^ increased progressively with age (Fig. 1). Consequently, the higher sensitivity of eGFR60 for identification of individuals with increased risk of death or CV event among participants ≥70 years of age was at the expense of decreased specificity due to increased numbers of false positives because of normal age-related decline in eGFR.

Variability of the KCD score reflects variability in eGFR measurement due to methodology and patient-specific factors. Given that a below-normal eGFR was more likely than an above-normal eGFR in the SCREEN-HF cohort, we considered the KCD limit of − 20 years to represent the lower limit of normal variation in KCD score for individuals without kidney disease. Thus, assuming a normal distribution for KCD score, the upper limit of normal variation in KCD score would be + 20 years, which was consistent with our data showing a KCD score ≥ 20 years (KCD20) was associated with increased risk of death or CV event. According to the full-age spectrum (FAS) equation [9], the lower reference eGFR (95% CI) for an individual aged 70 years is 56 ml/min/1.73 m^2^, which corresponds to a KCD score of 24 years. However, in contrast to the KCD score, the relationship between the lower reference eGFR from the FAS equation and health outcomes is unknown. Whereas the lower 95% CI of the FAS equation provides a single cut point, the KCD score provides a continuous age-adapted measure of kidney function that can be easily calculated and reported by the laboratory, analogous to the reporting of eGFR results. Moreover, in contrast to eGFRstep, the KCD score avoids the “birthday paradox” whereby CKD is “cured” when an individual reaches a specific age category [16].

It is of note that the 5482 healthy living potential kidney donors analysed by Pottel et al. [3] were selected by 12 different study groups, with variation between studies in mean GFR and 95% CI for specific age categories. The KCD score may assist in the definition of “normal” eGFR for age, and in the selection of living potential kidney donors.

By providing an age-adapted measure of kidney function, the KCD score may assist in patient education. Informing a patient that their kidney function is equivalent to that of an individual 20 or more years older than the patient’s age may be more informative for the patient than telling them their eGFR result in ml/min/1.73 m^2^, and may assist in improving patient compliance with therapy.

Our finding that addition of KCD20, eGFRstep or eGFR60 to a CV risk factor model failed to improve the AUC beyond that of the model based on CV risk factors alone was in agreement with previous studies [39], and the well-recognised insensitivity of ROC curve analysis to identify clinically important risk factors [40]. A meta-analysis of data from 637,315 participants showed that eGFR and albuminuria improved AUC when added to a CV risk factor model, although the increment in AUC was < 0.02 [19]. This small increment in AUC when kidney function parameters were added to a CV risk factor model reflected the extensive overlap between risk factors for CKD and CV disease, with age, hypertension, diabetes, obesity and smoking being major risk factors for both conditions [1].

Our study had a number of limitations. The SCREEN-HF cohort comprised volunteers (possible healthy volunteer bias) who were predominantly members of a health fund and, together with the inclusion criteria with respect to age ≥ 60 years and CV risk factors, may be cause for caution in the generalisation of our findings to the general community. However, the SCREEN-HF cohort was similar to the general Australian population aged ≥60 years. Of Australians aged 65–74 years, 53% have CV disease [41], 38.2% of men and 32.7% of women are obese [42], 17% have diabetes [43], 5% have AF [44], and 70% have hypertension [45]. Our findings are therefore likely to be applicable to the general Australian community. Although the SCREEN-HF cohort was limited to 3837 participants in this study, the inclusion criteria ensured sufficient events were observed during follow-up, although follow-up was relatively short. Another limitation was that we did not collect data for kidney outcomes such as end-stage kidney disease.

A further limitation of our study was that our analysis was based on a single eGFR measurement for each participant. However, any misclassification would have biased our results toward the null hypothesis. Moreover, most of the CKD Prognosis Consortium analyses of eGFR and risk of adverse events in both high-risk and general populations used as the reference group participants with only a single eGFR available [46]. Our equation for the calculation of KCD score was based on the meta-analysis of cross-sectional measured GFR reported by Pottel et al. [3]. Although eGFR calculated from the CKD-EPI equation may differ from measured GFR, the identification of individuals with increased risk of death or CV event by KCD20 in the SCREEN-HF cohort provided support for our use of CKD-EPI eGFR to calculate the KCD score.

We did not have urine data and therefore cannot report the proportion of participants with CKD according to urine albumin/creatinine ratio. Serum creatinine and eGFR are frequently measured as part of routine blood biochemistry, whereas the decision to measure albuminuria is usually based on an individual’s risk factor profile [47]. Previous studies reported most individuals with eGFR ≤60 ml/min/1.73 m^2^ did not have increased albuminuria [2, 48, 49], and eGFR ≤60 ml/min/1.73 m^2^ was associated with increased all-cause and cardiovascular mortality in the absence of increased albuminuria [2, 10]. However, increased albuminuria was a risk factor for all-cause mortality independent of eGFR [2, 20], and reduced eGFR with increased albuminuria was associated with increased risk for combined CV events and all-cause mortality [2, 48]. Thus, the threshold KCD score to define CKD may need to be modified if increased albuminuria is present. Certainly, an elevated KCD score should prompt measurement of albuminuria.

## Conclusions

In summary, the KCD score provided an age-adapted measure of kidney function that may assist patient education, and a KCD score ≥ 20 years (KCD20) provided an age-adapted criterion of eGFR-related increased risk of death or CV event. KCD20 was more sensitive than eGFRstep in identifying individuals aged ≥60 years with increased risk of death or CV event, and more sensitive than eGFR60 in identifying individuals aged 60–69 years with increased risk of death or CV event. Further studies that include the full age spectrum are required to examine the optimal KCD score cut point that identifies increased risk of death or CV event, and kidney events, associated with impaired kidney function, and whether the optimal KCD score cut point is similar for men and women.

## Supplementary Information

**Additional file 1.**

## Data Availability

The datasets used and/or analysed during the current study are available from the corresponding author on reasonable request.

## Abbreviations

AF: Atrial fibrillation
BMI: Body mass index
CKD: Chronic kidney disease
CKD-EPI: Chronic Kidney Disease Epidemiology Consortium
CV: Cardiovascular
eGFR: Estimated glomerular filtration rate
eGFRstep: The age-dependent stepped eGFR criteria of Delanaye et al. [10]
eGFR60: eGFR cut point of 60 ml/min/1.73 m^2^
FAS: Full-age spectrum
GFR: Glomerular filtration rate
HR: Hazard ratio
IQR: Interquartile range
KCD: Kidney age - Chronological age Difference
KCD20: Kidney age - Chronological age Difference score ≥ 20 years
ROC: Receiver operating characteristic
SCREEN-HF: SCReening Evaluation of the Evolution of New Heart Failure
