# Supplementary material for: Kidney age - chronological age difference (KCD) score provides an age-adapted measure of kidney function
Source: BMC Nephrol. 2021 Apr 26;22:152. doi: 10.1186/s12882-021-02324-y (PMC8077774; doi:10.1186/s12882-021-02324-y)
Supplement: Supplementary file 1 — Additional file 1. [file 12882_2021_2324_MOESM1_ESM.docx]

**Kidney age - Chronological age Difference (KCD) score: an age-adapted measure of kidney function**

Duncan J. Campbell, Jennifer M. Coller, Fei Fei Gong, Michele McGrady, Umberto Boffa, Louise Shiel, Danny Liew, Simon Stewart, Alice J. Owen, Henry Krum,

Christopher M. Reid, and David L. Prior

**Supplementary data**

**Table of contents**

**Table 1.** Participant outcomes.

**Table 2.** Sensitivities and specificities for the identification of men and women who experienced death or CV event during 5-year follow-up by KCD20, eGFRstep, and eGFR60 for all participants, and for participants aged 60-<70 years, 70-<80 years, and ≥80 years.

**Figure 1.** CONSORT flow diagram describing participant recruitment and follow-up in the SCREEN-HF study.

**Figure 2.** Body mass index according to categories of Kidney age-Chronological age Difference (KCD) score. Data shown as box and whisker plots, where the box indicates the median and interquartile range, the whiskers indicate the 10th and 90th percentiles, and individual data points indicate participants with body mass index outside these percentiles.

**Figure 3.** Calibration plots comparing model-based calculation of sex-specific 5-year absolute risk of death or cardiovascular (CV) event with mean Kaplan-Meier estimate of observed 5-year risk for deciles of absolute risk; models were based on CV risk factors alone (A), or together with Kidney age-Chronological age Difference score ≥20 years (KCD20, B), age-dependent stepped eGFR criteria of Delanaye et al. [1] (eGFRstep, C), or eGFR <60 ml/min/1.73 m^2^ (eGFR60, D). Brier scores, calculated from multivariable proportional hazards analysis, are shown with 95% confidence interval.

**Supplementary Table 1.** Participant outcomes.

___________________________________________________________________________

Outcome Total follow-up 5-year follow-up

Men Women Men Women

n 2096 1741 2096 1741

___________________________________________________________________________

Death or cardiovascular event 527 255 407 178

Death 189 108 141 73

Cardiovascular death 58 29 47 18

Non-cardiovascular death 131 79 94 55

Heart failure 97 64 70 40

Myocardial infarction 123 35 100 25

Stroke/transient ischaemic attack 89 72 71 56

Coronary revascularisation 213 51 181 37

___________________________________________________________________________

Cardiovascular events were incident myocardial infarction, heart failure, stroke/transient ischemic attack, and coronary revascularisation. Participants could have more than one cardiovascular event.

**Supplementary Table 2.** Sensitivities and specificities for the identification of men and women who experienced death or CV event during 5-year follow-up by KCD20, eGFRstep, and eGFR60 for all participants, and for participants aged 60-<70 years, 70-<80 years, and ≥80 years.

___________________________________________________________________________

Men Women

___________________________________________________________________________

Identification of death or CV event during 5-year follow-up by KCD20

All Participants

Sensitivity 23.8% 23.0%

Specificity 83.7% 82.1%

Participants aged 60-<70 years

Sensitivity 21.5% 27.8%

Specificity 85.9% 84.6%

Participants aged 70-<80 years

Sensitivity 23.9% 20.5%

Specificity 82.1% 80.6%

Participants aged ≥80 years

Sensitivity 29.0% 23.1%

Specificity 76.1% 73.6%

Identification of death or CV event during 5-year follow-up by eGFRstep

All Participants

Sensitivity 10.6% 11.2%

Specificity 94.6% 93.4%

Participants aged 60-<70 years

Sensitivity 7.0% 10.4%

Specificity 95.9% 94.4%

Participants aged 70-<80 years

Sensitivity 9.4% 6.4%

Specificity 95.0% 94.7%

Participants aged ≥80 years

Sensitivity 21.7% 19.2%

Specificity 81.2% 82.1%

Identification of death or CV event during 5-year follow-up by eGFR60

All Participants

Sensitivity 29.7% 32.0%

Specificity 82.8% 79.8%

Participants aged 60-<70 years

Sensitivity 17.1% 18.8%

Specificity 91.0% 89.0%

Participants aged 70-<80 years

Sensitivity 31.1% 28.2%

Specificity 76.7% 72.4%

Participants aged ≥80 years

Sensitivity 55.7% 50.0%

Specificity 57.3% 55.7%

___________________________________________________________________________

KCD20: Kidney age - Chronological age Difference score ≥20 years; eGFRstep: age-dependent stepped eGFR criteria of Delanaye et al. [1]; eGFR60: eGFR <60 ml/min/1.73 m^2^.

**Supplementary Fig. 1.** CONSORT flow diagram describing participant recruitment and follow-up in the SCREEN-HF study. Durations of follow-up for visits and phone review are shown as medians (interquartile range).

**Supplementary Fig. 2.** Body mass index according to categories of Kidney age-Chronological age Difference (KCD) score. Data shown as box and whisker plots, where the box indicates the median and interquartile range, the whiskers indicate the 10th and 90th percentiles, and individual data points indicate participants with body mass index outside these percentiles.

**Supplementary Fig. 3.** Calibration plots comparing model-based calculation of sex-specific 5-year absolute risk of death or cardiovascular (CV) event with mean Kaplan-Meier estimate of observed 5-year risk for deciles of absolute risk; models were based on CV risk factors alone (A), or together with Kidney age-Chronological age Difference score ≥20 years (KCD20, B), age-dependent stepped eGFR criteria of Delanaye et al. [1] (eGFRstep, C), or eGFR <60 ml/min/1.73 m^2^ (eGFR60, D). Brier scores, calculated from multivariable proportional hazards analysis, are shown with 95% confidence interval.

1. Delanaye P, Jager KJ, Bokenkamp A, Christensson A, Dubourg L, Eriksen BO, et al. CKD: a call for an age-adapted definition. J Am Soc Nephrol. 2019 Oct;30(10):1785-1805.
